# Low doses of neonicotinoid pesticides in food rewards impair short-term olfactory memory in foraging-age honeybees

**DOI:** 10.1038/srep15322

**Published:** 2015-10-19

**Authors:** Geraldine A. Wright, Samantha Softley, Helen Earnshaw

**Affiliations:** 1 Institute of Neuroscience, Faculty of Medical Sciences, Newcastle University, Newcastle upon Tyne, NE1 7RU, UK; 2 School of Biology, Faculty of Science, Agriculture, and Engineering, Newcastle University, Newcastle upon Tyne, NE1 7RU, UK

## Abstract

Neonicotinoids are often applied as systemic seed treatments to crops and have reported negative impact on pollinators when they appear in floral nectar and pollen. Recently, we found that bees in a two-choice assay prefer to consume solutions containing field-relevant doses of the neonicotinoid pesticides, imidacloprid (IMD) and thiamethoxam (TMX), to sucrose alone. This suggests that neonicotinoids enhance the rewarding properties of sucrose and that low, acute doses could improve learning and memory in bees. To test this, we trained foraging-age honeybees to learn to associate floral scent with a reward containing nectar-relevant concentrations of IMD and TMX and tested their short (STM) and long-term (LTM) olfactory memories. Contrary to our predictions, we found that none of the solutions enhanced the rate of olfactory learning and some of them impaired it. In particular, the effect of 10 nM IMD was observed by the second conditioning trial and persisted 24 h later. In most other groups, exposure to IMD and TMX affected STM but not LTM. Our data show that negative impacts of low doses of IMD and TMX do not require long-term exposure and suggest that impacts of neonicotinoids on olfaction are greater than their effects on rewarding memories.

Neonicotinoid compounds applied as systemic seed treatments to crops are some of the most commonly used insecticides in the world^1^,^2^. Systemic administration of pesticides to seeds causes pesticides to permeate plant tissues including floral nectar and pollen^3^. However, the extent to which pesticide-contaminated nectar and pollen affect insect pollinators is controversial. Several studies have shown that sub-lethal doses of neonicotinoids disrupt important behaviours including navigation^4^,^5^, learning and memory^6^,^7^,^8^,^9^, and motor function^10^,^11^ in honeybees. However, these studies have often been performed using concentrations of neonicotinoids greater than the amount found in the nectar and pollen of seed-treated crops^12^,^13^.

Recently, we observed that honeybees presented with a choice of two solutions prefer to consume sucrose laced with nectar-relevant doses of the neonicotinoid pesticides, imidacloprid (IMD) and thiamethoxam (TMX), to sucrose alone^14^. We also found that the bees’ mouthparts gustatory sensilla do not respond to these compounds when they are present in sucrose solutions. We reasoned that bees could not taste nectar-relevant concentrations of IMD and TMX, but instead consumed more of these solutions because of a pharmacological effect of these compounds on the bee’s brains. Neonicotinoids are agonists of insect nicotinergic acetylcholine receptors (nAChRs)^15^, and their activation at very low doses could enhance neurotransmission in the neurons involved encoding rewarding memories of food^16^. For this reason, we predicted that bees exposed to IMD and TMX in nectar rewards during an olfactory learning task should exhibit enhanced learning and memory.

In contrast, however, several previous studies have shown that honeybees exposed to neonicotinoids prior to learning and memory tasks exhibit slower rates of learning and poor memory formation^6^,^7^,^8^,^9^. Most studies of the impact of neonicotinoids on bee learning expose bees to neonicotinoids chronically for several days prior to olfactory conditioning of the proboscis extension response (PER)^6^,^7^,^8^,^9^ or administer one dose within an hour before conditioning^6^,^17^. We could find only one study in which bees were trained with food containing neonicotinoids during conditioning, and this was for a 1-trial olfactory learning task using a very high dose that did not examine the impact on learning^6^. Additionally, most previous work has used concentrations that exceed levels found in the nectar of pesticide-treated plants. None have examined learning performance in a task with repeated conditioning trials that mirrors what bees would experience while foraging on the nectar of seed-treated plants.

Based on Kessler *et al.* (2015)^14^, we predicted that bees fed with IMD and TMX should exhibit faster rates of learning and longer lasting memories if neonicotinoids experienced in nectar increased the value of the reward during a learning task. To test this, we examined how nectar-relevant concentrations of IMD and TMX in sucrose rewards affected the rate of learning during olfactory conditioning of the PER. We trained bees using two conditioning protocols (massed and spaced) to measure their effects on the honeybee’s short (STM) and long-term (LTM) appetitive memories. Massed conditioning (30 s inter-trial interval) was used to examine how IMD and TMX would affect learning as bees might experience it during foraging. Spaced conditioning (5 min inter-trial interval) was used to determine the extent to which IMD and TMX affected the formation of LTM.

## Results

### IMD in food rewards impairs olfactory learning

We first measured the proportion of bees that failed to exhibit a learned response on any of the trials during conditioning. Bees fed with sucrose solutions containing 10 nM IMD during massed or spaced conditioning were less likely to learn the task than the control bees (Table 1, massed: lreg, χ_3_^2^ = 9.48, P = 0.023; spaced: lreg, χ_3_^2^ = 10.9, P = 0.012). TMX did not significantly affect the number of bees that failed to exhibit learned responses (Table 1, massed: lreg, χ_3_^2^ = 6.41, P = 0.093; spaced: lreg, χ_3_^2^ = 3.26, P = 0.353).

**Figure 1:**
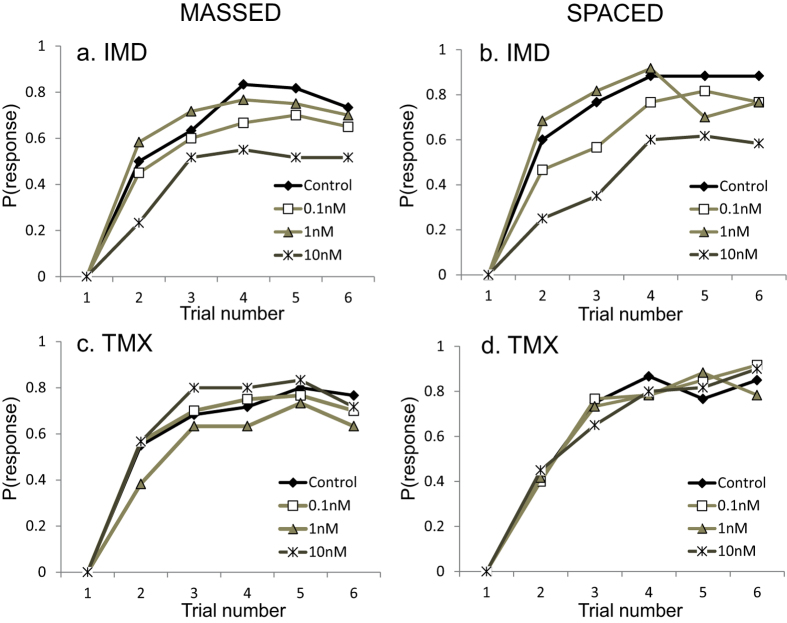
Bees rewarded with 1 M sucrose solution containing IMD during massed (a) and spaced (b) conditioning had a slower rate of learning. Bees rewarded with TMX solutions were largely unaffected during massed (**c**) and spaced (**d**) conditioning. The y-axis represents the probability of eliciting a conditioned PER to the odour prior to food presentation. Each treatment group for each neonicotinoid contained 60 responding subjects, therefore the overall sample size consisted of 480 honeybees.

Providing honeybees with sucrose solution containing IMD or TMX as a reward did not enhance learning in either the massed or spaced learning tasks (Fig. 1). To show this, we separately analysed the rate of learning of the bees that exhibited conditioned PER on at least one trial during the task (Fig. 1). Bees rewarded with sucrose containing IMD during both massed and spaced learning were less likely to learn to associate the odour with food (Fig. 1a,b, massed: repeated-measures lreg, χ_3_^2^ = 29.9, P < 0.001; spaced: repeated-measures lreg, χ_3_^2^ = 56.8, P < 0.001). The impact of IMD on learning manifested as early as the second conditioning trial; approximately half as many (massed: 23%, spaced: 25%) of the bees responded on the 2^nd^ trial in the 10 nM IMD group compared to the control (massed: P = 0.002; spaced: P < 0.001).

Bees fed with 1 nM TMX during massed conditioning had a slower rate of learning than the control (Fig. 1c; repeated-measures lreg, χ_3_^2^ = 8.30, P = 0.040). TMX did not affect learning in the spaced conditioning task (Fig. 1d; repeated-measures lreg, χ_3_^2^ = 0.440, P = 0.931).

### IMD and TMX impair short-term olfactory memory

**Figure 2:**
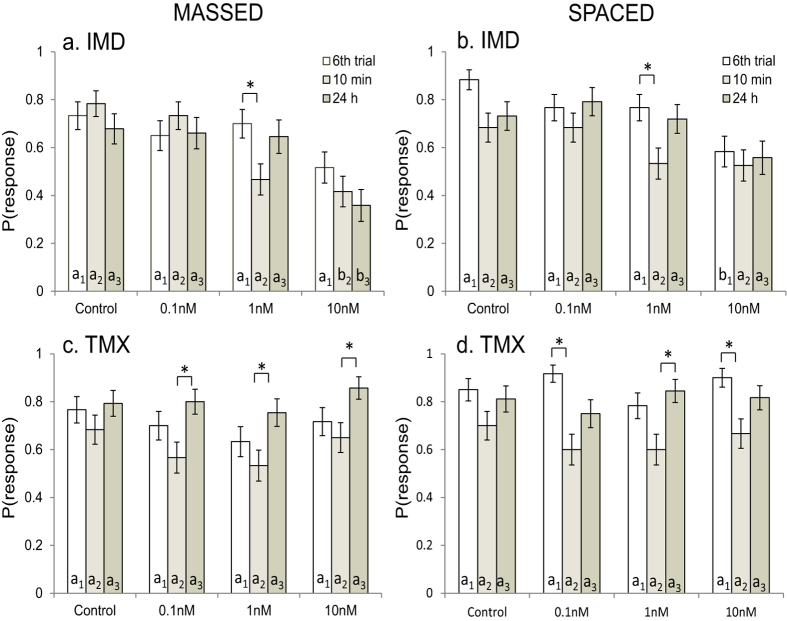
Comparisons of the bees’ conditioned PER responses on the 6^th^ conditioning trial to their responses at 10 min (STM) and 24 h (LTM) after training. Bees fed with rewards containing 10 nM IMD were less likely to respond overall during massed (**a**) and spaced (**b**) conditioning. Bees fed with TMX were least likely to respond during the 10 min test and most likely to respond during the 24 h test during both massed (**c**) and spaced (**d**) conditioning. Error bars are + SE. Letters indicate comparisons of the control group to each treatment group (subscripts: 1 = 6^th^ trial, 2 = 10 min, 3 = 24 h). *indicates P < 0.003 (Bonferroni-adjusted critical value) for comparisons within treatment groups. Each treatment group contained 60 responding subjects in the STM test at 10 min. The sample size for the 24 h test changed overnight because some bees died. For the massed trained group at the 24 h test, the sample sizes were as follows: IMD: control = 56, 0.1 nM = 53, 1 nM = 48, 10 nM = 53; TMX: control = 58, 0.1 nM = 60, 1 nM = 57, 10 nM = 56. For the spaced-trained group at the 24 h test, the sample sizes were as follows: IMD: control = 56, 0.1 nM = 48, 1 nM = 57, 10 nM = 52; TMX: control = 53, 0.1 nM = 58, 1 nM = 58, 10 nM = 60.

We also predicted that if IMD and TMX enhanced the rewarding properties of the food during conditioning, bees should respond more to the conditioned odour during the memory tests at 10 min and 24 h than the control. Memory was mainly tested via comparison of the responses of bees on the 6^th^ trial to their responses at 10 min (STM) and 24 h (LTM) after the training period, but we also compared performance relative to the control. The responses of bees fed with IMD during massed conditioning depended both on the dose of IMD and the time of the test (Fig. 2a, repeated-measures lreg, US x test time: χ_6_^2^ = 14.5, P = 0.024). Spaced-conditioned bees responded less when fed with 10 nM IMD (Fig. 2b, repeated-measures lreg, US: χ_3_^2^ = 20.1, P < 0.001), but on average, the responses were lowest during the STM test (repeated-measures lreg, time point: χ_2_^2^ = 19.2, P < 0.001). In particular, the bees fed 10 nM IMD had low performance during both tests whereas those fed 1 nM IMD had poor performance only during the 10 min test for STM.

Bees fed TMX were less likely to respond to the test odour at 10 min than at 24 h (Fig. 2c,d; massed: repeated-measures lreg, test time: χ_2_^2^ = 26.1, P < 0.001; spaced: repeated-measures lreg, test time: χ_3_^2^ = 45.9, P < 0.001). On average, the responses at each time point of the bees fed with TMX were not different to the controls (Fig. 2c,d). However, comparisons within groups revealed that bees conditioned with TMX in rewards were more likely to respond during the LTM test than the STM test.

## Discussion

The premise of these experiments was to test whether low, field-relevant doses of the neonicotinoids, IMD and TMX, enhanced learning and memory when they were present in food rewards during olfactory conditioning. Contrary to our predictions, we found that IMD and TMX in some cases impaired learning and consistently reduced performance during the STM test. Performance during the 24 h test for LTM was largely unaffected.

Our data show that the impact of neonicotinoids on olfactory learning depends on the dose but not on the period of exposure prior to the learning task. Several groups have used olfactory PER conditioning to show that long-term exposure to neonicotinoids such as IMD impairs learning and memory^6^,^7^,^8^,^9^,^17^,^18^,^19^,^20^. ‘Impairment’ as previously defined is largely characterized by fewer bees exhibiting conditioned responses and fewer responding consistently during training. The only previous study providing bees with neonicotinoids in reward during conditioning administered 12 ng/bee IMD during a single conditioning trial^6^. Using a test for olfactory memory, they reported that 70% fewer bees recognized the conditioned odour 24 h later. Our experiments establish that long-term exposure is not necessary to observe a reduction in the responses of a population of bees during olfactory learning and afterwards in memory tests. We found that an acute dose of 6.12 pg/bee IMD (i.e. six 0.4 μl droplets of 10 nM IMD-laced reward) or 0.69 pg/bee TMX (i.e. six 0.4 μl droplets of 1 nM TMX) experienced during acquisition was sufficient to reduce the rate of learning. This effect occurred within 30 s of the consumption of the first food reward in the massed conditioning experiments. If bees could taste the pesticides, the reduced responding during conditioning could be accounted for by the fact that they were repelled by it and were learning to avoid odours associated with pesticide laced solution^21^. However, we established previously that honeybees cannot taste IMD and TMX in the sucrose solution^14^. For this reason, our experiments indicate that IMD gets across the blood-brain barrier very quickly to cause an observable change in the number of bees that respond during conditioning due to its direct action on the brain.

The most robust effect of IMD or TMX exposure we found was a reduction in the number of responses during the 10 min STM test. The metabolism of neonicotinoids takes bees several hours^22^; the STM test would have been the period of greatest exposure to all of the pesticide doses in the experiments. In contrast, however, concentrations of 1 nM (0.612 pg/bee) doses or less of IMD or TMX had no negative effects on olfactory LTM memory in honeybees. It is possible that TMX may have even slightly enhanced performance during the LTM test, but our assay was not sensitive enough to measure it. Honeybees metabolise IMD rapidly, and a dose as high as 50 μg/bee is completely metabolised in 24 h^22^. For this reason, we suspect that the bees’ poor performance in the learning and STM test was a result of a direct influence of IMD or TMX on nAChRs in the olfactory system which may have occluded an effect on the areas of the brain involved in reward.

Both IMD and TMX also have compound-specific pharmacological effects on nAChRs, and these receptors have specific distributions in bee’s neuropil^23^,^24^. Previous studies in the honeybee have shown that antennal lobe neurons responsible for encoding information about odours express 4 different nAChR subunits (a2, a7, a8, b1) whereas Kenyon cells express 3 (a2, a8, b1)^24^. Other neurons in the honeybee brain have not yet been examined in detail, but could express any combination of the subunits, as all 11 are expressed in the bee brain^24^. The Kenyon cells and a subset of the antennal lobe neurons have been described as housing fast desensitizing nAChRs; Kenyon cells exhibit fast neuronal inactivation at relatively low doses of IMD^16^,^24^. We predict that neuronal activation of antennal lobe neurons and/or Kenyon cells accounts for the reduced performance of bees given 10 nM IMD in reward in our experiments.

Neurons in the insect olfactory system are primarily cholinergic^25^ and inactivation of neurons or desensitization of the receptors by IMD^16^,^24^ could rapidly lead to disruption of neurotransmission. Studies that have administered IMD prior to testing bees in a gustatory assay have shown that bees are less sensitive to sucrose applied to their antennae^18^,^26^ and are more apt to habituate the PER when the antennae are stimulated with sucrose^27^,^28^ supporting the idea that exposure to IMD disrupts sensory function in gustation as in olfaction^24^. Another way that IMD exposure could affect olfaction is by altering the activity of neurons in the antennal lobe or the Kenyon cells of the mushroom bodies. This would cause changes to odour perception and hence odour recognition. For example, a recent study in the moth, *Agrotis ipsilon*, found that low doses (0.25 ng) of clothianidin in food made male moths less likely to recognize a female pheromone, but that slightly greater doses (10 ng) increased the males’ recognition and hence attraction to the pheromone^29^. Coding of odours is complex and involves precise excitation and inhibition in the antennal lobe and Kenyon cells^30^,^31^; slight changes in the way the antennal lobe network is balanced could influence olfactory perception and hence odour recognition^32^.

The fact that we did not observe an obvious enhancement in olfactory learning or memory as we predicted could also be a result of the doses we used. The concentrations with the greatest effect on honeybees in Kessler *et al.* (2015) were 100 nM IMD and TMX^14^. Concentrations as high as 10 nM IMD or TMX are rarely reported from the nectar of seed-treated crops^3^,^33^,^34^, but concentrations as high as 40 nM IMD and TMX have been obtained from the nectar of orange trees that have been sprayed while in flower or soil-treated cucurbit crops^35^,^36^. Overall, the lowest concentrations we tested (e.g. 0.1–1 nM) affected STM, but had no lasting impact on LTM. Bees are likely to visit several hundred flowers during foraging, which could increase the dose they would be exposed to even if nectar concentrations were as low as 1 nM. For example, if a bee fed on nectar laced with 1 nM IMD, it would need to collect sixty 0.4 μl droplets (24 μl) to achieve a 6 pg/bee dose. This quantity is in the range of the volume of the honeybee’s crop (i.e. honey stomach), but they also hold much of the nectar they collect in their crop to bring it back to the colony as nectar.

Our data show that IMD and TMX concentrations in nectar <1 nM are not likely to have appreciable lasting effects on honeybee olfactory learning and memory when experienced during a short foraging bout. As predicted by Kessler *et al.* (2015)^14^, IMD and TMX could, however, affect other forms of learning and memory such as spatial memory and may have stronger effects on other bee species. Exposure to concentrations greater than 1 nM or that lasts several days and that leads to an accumulation of IMD or TMX, however, would ultimately impact neuronal function and lead to poor foraging performance in an olfactory learning task.

## Materials and Methods

### Honeybees

Foraging adult worker honeybees (*Apis mellifera* var. Buckfast) were obtained from the UK’s National Bee Unit (Sand Hutton, Yorkshire); colonies were maintained outdoors at Newcastle University. The bees were collected from the entrance of the hive in glass vials, cold anaesthetised for 2–3 min on ice, and then restrained in modified plastic harness (Fig. 1b). After harnessing, bees were fed with 15 μl of 0.7 M sucrose solution using a 2 ml Gilmont micrometer syringe (GS-1200), and left in a humidified box at RT for ~18–22 h. The next day, the antennae of each subject were stimulated with a droplet of 0.7 M sucrose solution to provoke the proboscis extension reflex (PER); if a bee did not respond by extending its proboscis, it was not used in the experiments.

### Conditioning procedure and stimuli

Honeybees were trained using a procedure for olfactory conditioning of the proboscis extension reflex^37^. The conditioned (CS) and unconditioned stimuli (US) were presented on a massed schedule (30 s inter-trial interval) or a spaced schedule (5 min inter-trial interval)^38^. The conditioned stimulus was the odour, 1-hexanol (99.8% purity; Sigma-Aldrich, St Louis, MO), presented for 4 s duration, and the unconditioned stimulus was a reward of 0.4 μl of treatment solution. The odour stimulus arose from a 3 μl aliquot applied to a strip of filter paper placed within a glass tube and attached to controlled air supply (the arena and training apparatus are previously described in Wright *et al.* (2007)^39^. The unconditioned stimulus was one of the following solutions: 0.7 M sucrose (control), or 0.7 M sucrose containing 0.1 nM, 1 nM, 10 nM of imidacloprid or thiamethoxam. (A 10 nM solution of IMD equates to 2.55 pg/μl; a 10 nM solution of TMX equates to 2.91 pg/μl. The entire dose received during conditioning for bees trained with 10 nM IMD would be 6.12 pg/bee). Imidacloprid and thiamethoxam were obtained in dry powder form (Pestanal, Sigma-Aldrich); solutions were made by directly dissolving the powder into 0.7 M sucrose to make a stock solution that diluted to the correct concentration using 0.7 M sucrose solution. Bees that failed to exhibit PER when stimulated with 0.7 M sucrose solution applied to the antennae were omitted from training; likewise, bees that responded to the conditioned stimulus prior to conditioning were removed from the experiment. Each subject received 6 conditioning trials. After conditioning, each bee was tested with the conditioned stimulus and a novel odour (2-octanone) at 10 min and 24 h. The order of presentation of the test odours was randomized across subjects, and each test was presented with a 3–5 min interval between each test. The 10 min test was performed to assess short-term memory (STM) and the 24 h test was performed to test early long-term memory (LTM)^38^. The bees were fed 15 μl of 0.7 M sucrose solution within 30 min of the 10 min test and left in a humidified box at RT to ensure survival to the 24 h test.

### Statistics

Analyses were performed using IBM SPSS v.21. The response of each subject to the odour stimulus during conditioning and testing was scored as a binary response (proboscis extension). The conditioning data were partitioned into ‘responding’ and ‘non-responding’ bees; non-responders were subjects that did not exhibit conditioned PER on any of the trials. Generalized linear models for logistic regression (lreg) were used to analyze the proportion of bees that did not respond during conditioning. For the conditioning and memory test data, we partitioned out only the animals that responded during conditioning. The acquisition curves and the tests were analyzed using repeated-measures lreg (generalized estimating equations in SPSS). The first training trial was excluded from the analysis to facilitate model fit. Least squares *post-hoc* tests were performed for pair-wise comparisons against the control for the learned response data and the test data; critical values were Bonferroni-adjusted for specific hypotheses.

## Additional Information

**How to cite this article**: Wright, G. A. *et al.* Low doses of neonicotinoid pesticides in food rewards impair short-term olfactory memory in foraging-age honeybees. *Sci. Rep.* **5**, 15322; doi: 10.1038/srep15322 (2015).

## Figures and Tables

**Table 1:** Relative proportion of bees that failed to respond on any trials during the conditioning experiments in Fig. 1.

Massed	IMD	TMX
Control	0.31 (87)	0.05 (63)
0.1 nM	0.39 (98)	0.17 (70)
1 nM	0.39 (99)	0.20 (75)
10 nM	0.51* (124)	0.10 (67)
**Spaced**	**IMD**	**TMX**
Control	0.26 (81)	0.09 (66)
0.1 nM	0.29 (84)	0.05 (63)
1 nM	0.24 (79)	0.03 (62)
10 nM	0.44* (107)	0.10 (67)

Number in parentheses is the total sample size for each group including responding and non-responding bees.

^*^indicates P < 0.05 for comparisons with control.
